# Nose-to-Brain Delivery of Biomimetic Nanoparticles for Glioblastoma Targeted Therapy

**DOI:** 10.1021/acsami.4c16837

**Published:** 2024-12-18

**Authors:** Natália Noronha Ferreira, Celisnolia Morais Leite, Natália Sanchez Moreno, Renata Rank Miranda, Paula Maria Pincela Lins, Camila Fernanda Rodero, Edilson de Oliveira Junior, Eliana Martins Lima, Rui M. Reis, Valtencir Zucolotto

**Affiliations:** † Nanomedicine and Nanotoxicology Group, Physics Institute of São Carlos, São Paulo University, Avenida Trabalhador São Carlense, 400, São Carlos, SP 13560-970, Brazil; ‡ Hasselt University, Faculty of Medicine and Life Sciences, Biomedical Research Institute (BIOMED), Agoralaan, 3590 Diepenbeek, Belgium; § Laboratório de Nanotecnologia Farmacêutica e Sistemas de Liberação de Fármacos, FarmaTec, Faculdade de Farmácia, Universidade Federal de Goiás − UFG, 5a Avenida c/Rua 240 s/n, Praça Universitária, Goiânia, GO 74605-170, Brazil; d Molecular Oncology Research Center, Barretos Cancer Hospital, Rua Antenor Duarte Villela, 1331, Barretos, SP 14784-400, Brazil; e Life and Health Sciences Research Institute (ICVS), School of Medicine, University of Minho, Campus de Gualtar, Braga 4710-057, Portugal

**Keywords:** biomimetic delivery systems, Temozolomide, nanotechnology, PLGA-based nanoparticles, glioblastoma treatment, homotypic recognition, nose-to-brain delivery

## Abstract

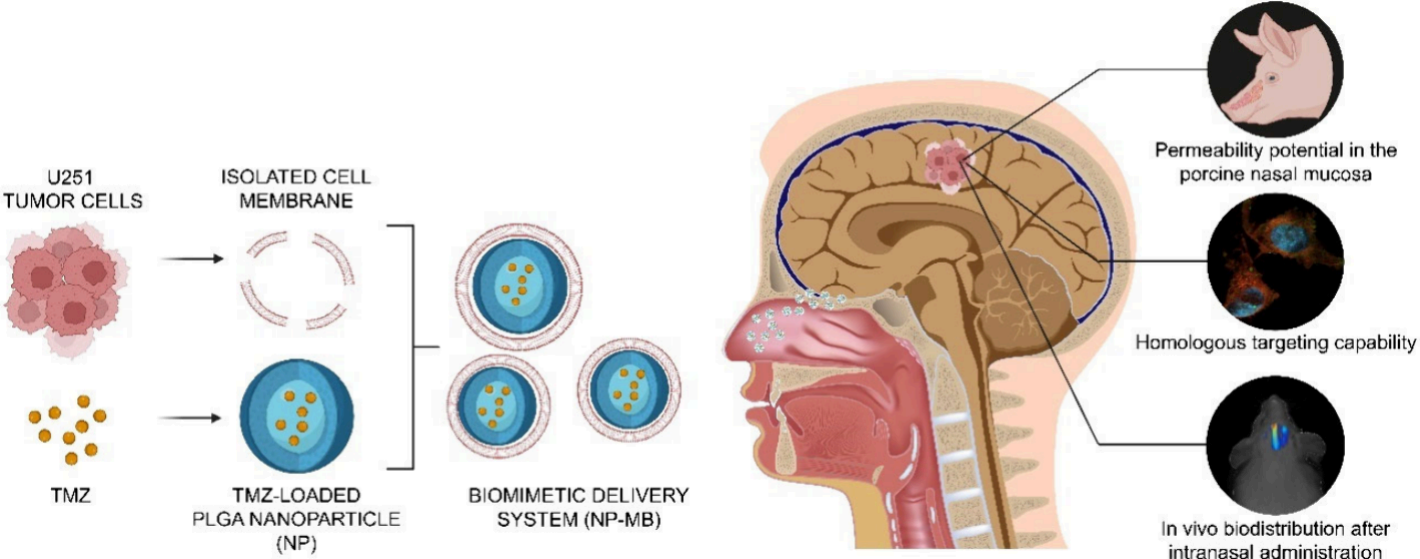

Glioblastoma (GBM) is an extremely aggressive form of brain cancer that remains challenging to treat, especially owing to the lack of effective targeting and drug delivery concerns. Due to its anatomical advantages, the nose-to-brain strategy is an interesting route for drug delivery. Nanoengineering has provided technological tools and innovative strategies to overcome biotechnological limitations, which is promising for improving the effectiveness of conventional therapies. Herein, we designed a biomimetic multifunctional nanostructure produced by polymeric poly(d,l-lactic-*co*-glycolic) acid (PLGA) core loaded with Temozolomide (TMZ) coated with cell membrane isolated from glioma cancer cells. The developed nanostructures (NP-MB) were fully characterized, and their biological performance was investigated extensively. The results indicate that NP-MB could control TMZ release and promote TMZ permeation in the *ex vivo* nasal porcine mucosa. The higher cytotoxicity of NP-MB in different glioma cell lines, particularly against U251 cells, reinforces their potential for homotypic targeting. The chicken chorioallantoic membrane assay revealed a tumor size reduction and antiangiogenic activity. *In vivo* biodistribution studies showed that NP-MB effectively reaches the brain following nasal administration. These findings suggest that NP-MB holds promise as a biomimetic nanoplatform for effective targeting and homotypic recognition in GBM therapy with high potential for clinical translation.

## 1 Introduction

Glioblastoma (GBM) is a prevalent malignant brain tumor with a high recurrence and mortality rate and an overall median survival rate of no longer than two years.^1^ Currently, surgical resection, radiotherapy, chemical therapy, and immunotherapy have been used alone or associated as standard treatments for GBM, where the Temozolomide (TMZ) prodrug is the gold standard chemotherapeutic.^2,3^ However, radiation and chemical therapy lead to rather limited results and generally trigger severe side effects,^4^ which may affect treatment success rates and the quality of life of patients.

Limited prognosis is usually related to tumor heterogeneity at the molecular and cellular level, drug resistance, and insufficient brain drug delivery owing to restricted access to tumors imposed by the blood–brain barrier (BBB). Although TMZ is able to cross the BBB, as for several systemically administered drugs, the dosage that potentially reaches the tumor site in the CNS is less than 1% of the total received, regardless of its BBB permeability potential.^3^ Reports show that the BBB, together with the blood–brain–tumor barrier (BBTB), prevents the access of over 98% of therapeutic agents to the brain tumor sites. Hence, a major obstacle to novel chemotherapeutic strategies for GBM and other malignant brain tumors is effective targeting and drug delivery.^3^

Recent studies have shown that nose-to-brain transport is an alternative route to enhance brain bioavailability because this administration route efficiently provides direct delivery through extracellular diffusion and olfactory or trigeminal neural pathways, bypassing the BBB.^5^ However, it is important to consider that this strategy presents certain limitations.^6,7^ For instance, owing to the small volume of the nasal cavity, large doses cannot be administered (the maximum dosing volume in humans is 0.4 mL). Another crucial factor is the presence of metabolic enzymes in mammalian olfactory mucosa. In addition, drug permeability and short drug residence time in the nasal epithelium owing to mucociliary clearance should be considered.^6^

Advances in the engineering of nanomaterials and their transposition to medical applications have provided potential strategies for effectively improving clinical outcomes. A major feature of nanotechnology is to provide drug protection, maintain drug stability and molecular configuration, enhance direct transport to the CNS, improve uptake by the olfactory mucosa, and provide direct access to the CNS.^8,9^

Over the past decade, notable advances in nanomedicine have enabled the development of multifunctional nanotherapeutics.^10^ Among these innovations, the use of natural cell membrane-coating technology for nanoparticles (NP) has garnered attention.^11−14^ This technique uses a core–shell structure in which a drug-loaded NP is coated with cell membrane vesicles and isolated from a given tissue or cell culture. Cell membranes obtained from tumor cells are important for cancer therapy, because they preserve the biological features of their source cancer cells. Therefore, these bioinspired and biomimetic NPs may benefit from their homologous binding and natural immune-evading properties, make them ideal nanoplatforms for precise drug delivery.^4,15^ The qualification of these systems to provide personalized therapy has been demonstrated using *in vitro* and *in vivo* protocols.^16−18^

In this study, we proposed combined strategies to enhance the efficiency of GBM treatment by integrating a novel bioinspired drug delivery system allied with nose-to-brain transport. The developed TMZ loading PLGA NP coated with isolated U251 glioma cell membranes (NP-MB) was extensively characterized in terms of drug-loading efficiency, size, PDI, surface charge, morphology, and stability. The ability of the nanostructure to modulate drug release properties and permeation patterns was assessed. The biological performances of NP and NP-MB were investigated using *in vitro*, *ex vivo*, and *in vivo* protocols. Overall, the developed NP-MB represents a promising nanoplatform for homotypic recognition, improved biological response, and a valuable novel and effective therapeutic opportunity for GBM treatment via a nose-to-brain delivery route.

## 2 Results and Discussion

Biomimetic designs can endow nanoparticles with complex functionalities, thereby enhancing their biological capabilities at the nanobiointerface. In this study, we describe a novel nanostructured biomimetic and bioinspired system to improve GBM treatment using homotypic targeting associated with the nose-to-brain pathway. TMZ, a gold standard treatment for GBM, is one of the most common antiglioma agents, especially because of its ability to penetrate the BBB.^19^ However, due to its stability limitations and regimen of administration, the inclusion of TMZ into biomimetic delivery systems, allowing the possibility of exploiting nose-to-brain administration, appears to be a promising way to improve therapeutic outcomes by increasing brain bioavailability and providing homotypic recognition by tumor cells.

**Figure 1:**
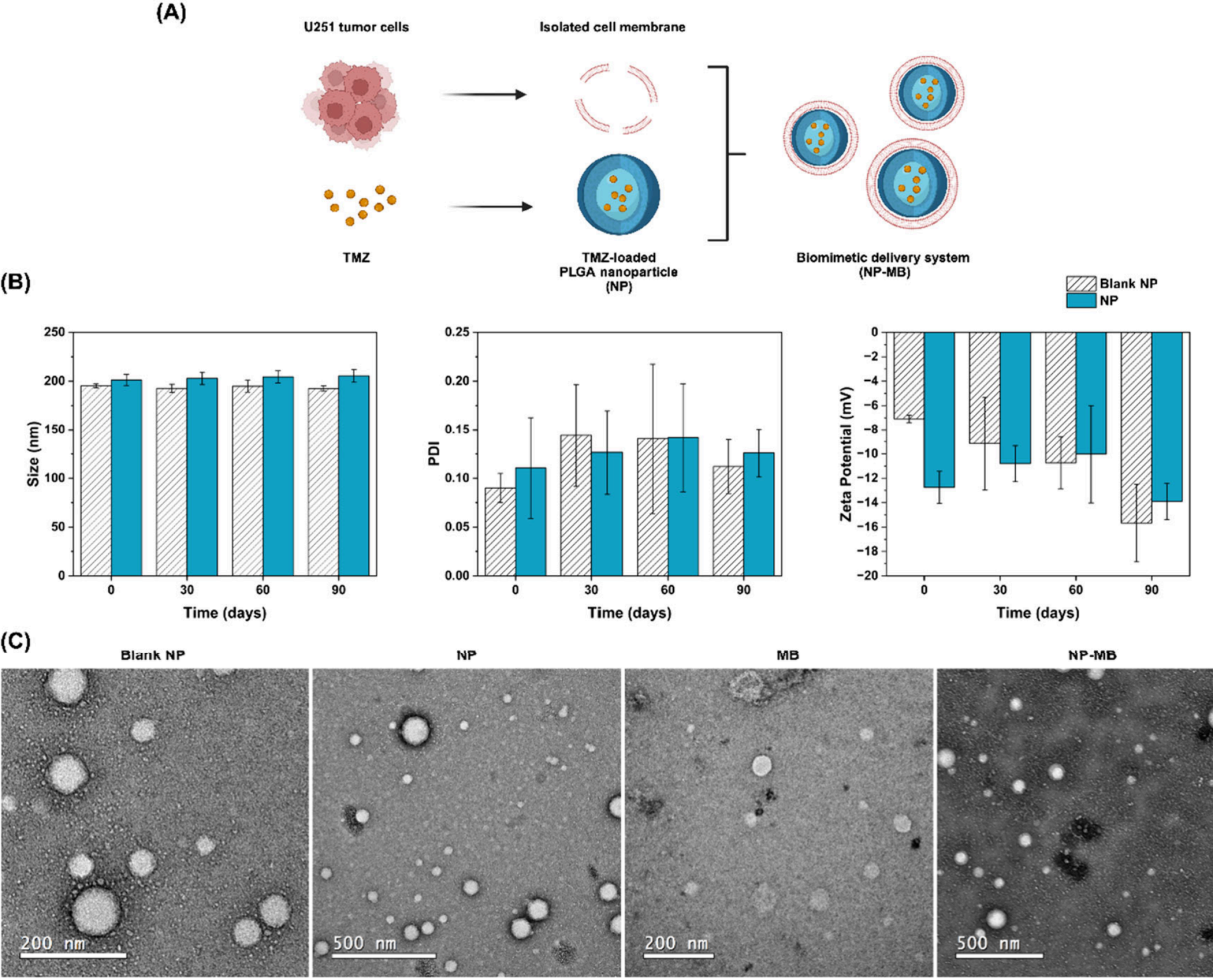
Development and characterization of NP-MB. (A) Schematic representation of NP and NP-MB development. Created with BioRender.com. (B) Evaluation of nanosystems Blank NP (uncolored plots) and NP (blue plots) stability in terms of size (nm), PDI, and zeta potential (mV). Statistical one-way ANOVA analysis was applied to identify differences over time *p* < 0.05. (C) Morphological analysis of nanostructures. Representative images of blank NP, NP, isolated membrane (MB) and NP-MB were recorded using negative-staining transmission electron microscopy (TEM) JEM-2100-JEOL 200 with a LaB6 source operating at an acceleration voltage of 200 Kv.

To study the biological performance of NP coating with an isolated tumor cell membrane, especially in terms of target delivery and homotypic recognition of GBM cells, we first prepared TMZ-loaded PLGA NP by using the double emulsion solvent evaporation method. Furthermore, the isolated U251 MB was coated onto the developed NP to compose the biomimetic NP-MB using a sonication bath, according to previously studied and optimized conditions.^20^ A schematic of the NP and NP-MB synthesis procedures is shown in Figure 1A.

### 2.1 Physicochemical Characterization

Physicochemical characterization of nanostructures is an important step in nanotechnology development, contributing to the understanding of their functional capabilities.^21^ The mean size, polydispersity index (PDI), zeta potential (ZP), mode, and concentration results are listed in Table 1.

**Table 1:** Nanostructure Characterization^a^

	Dynamic Light Scattering (DLS)			Nanotracking analysis (NTA)		
	Size (nm)	PDI	Zeta Potential (mV)	Mean(nm)	Mode (nm)	Concentration (particles/mL)
**Blank NP**	257 ± 22	0.10 ± 0.03	–18 ± 3	217 ± 25	189 ± 12	6 × 10^11^ ± 2 × 10^10^
**NP**	245 ± 16	0.08 ± 0.01	–16 ± 2	220 ± 40	202 ± 10	7 × 10^11^ ± 2 × 10^10^
**NP-MB**	260 ± 60	0.29 ± 0.05	–13 ± 1	154 ± 17	195 ± 25	3 × 10^11^ ± 5 × 10^9^

a Mean size, PDI, and ZP data from DLS analysis; mean, mode, and particle concentration from NTA analysis of developed blank NP, NP, and NP-MB. Data represent the average of at least 3 measurements (*n* = 3) and standard deviation. Statistical analysis using one-way ANOVA with Tukey’s comparisons was applied to identify differences between blank NP (control) and NP/NP-MB (*p* < 0.05).

Our data showed that both nanostructures (blank NP and NP) had a mean size of approximately 200–250 nm measured by dynamic light scattering (DLS) or nanotracking analysis (NTA) (Table 1). The PDI revealed the formation of a homogeneous population because the acquired values were less than 0.2.^22^ ZP was negative at approximately −15 mV for both blank NP and NP.

Considering the proposed coating procedure using isolated U251 cell membranes, NP stability should be evaluated to predict the colloidal behavior from the synthesis procedure to MB functionalization. These data represent an important concern that limits the application of nanostructures in clinical practice.^23^ The projection of stability over time can predict alterations that may affect biological performance *in vitro*. Therefore, blank NP and NP were frequently monitored for three months in terms of size, PDI, and ZP. The results are displayed in Figure 1B and show no significant changes in size or PDI during the analyzed period. A significant change was observed in the ZP value of empty NP from the initial to the 90th-d analysis. However, no significant changes in ZP were observed for NP after 90 d. These data suggest that the TMZ drug encapsulated in the nanostructure may have provided additional stability in terms of the ZP.

After the coating procedure using cell membranes isolated from U251 tumor cells (NP-MB), the size of the nanoparticles did not show significant alterations, with a mean size of approximately 260 nm, as measured by DLS. However, the PDI data depicted a more heterogeneous population due to the membrane PDI contribution (the PDI measured for isolated MB was 0.57 ± 0.12). ZP also showed a significant change as the values became closer to ZP of the isolated membrane (−10 ± 0.06 mV).

Negative-staining transmission electron microscopy (TEM) and cryo-TEM were used to investigate the nanostructure morphology. The image depicts spherical structures and reinforces the detected change in polydispersity (from blank NP to NP-MB) caused by the coating procedure for applying isolated MB. The recorded images showed no significant sites of particle or organic material aggregation (Figure 1C and Figure S1).

### 2.2 Protein Corona Formation

The biological performance of nanostructured drug delivery systems is drastically affected by several factors following *in vivo* administration. One of the most important effects are their interaction with proteins present in different biological fluids. This process, known as protein corona (PC) formation, refers to the absorption or binding of different proteins to the NP and NP-MB surfaces, which can affect the colloidal stability, drug release, mucoadhesive or mucopenetrating properties, and targeting ability.^24^ Proteins with a higher affinity for nanostructures can easily bind or interact with the NP surface and instantaneously constitute the hard corona. In contrast, low-affinity proteins gradually form at soft corona through dynamic processes.^25^

**Figure 2:**
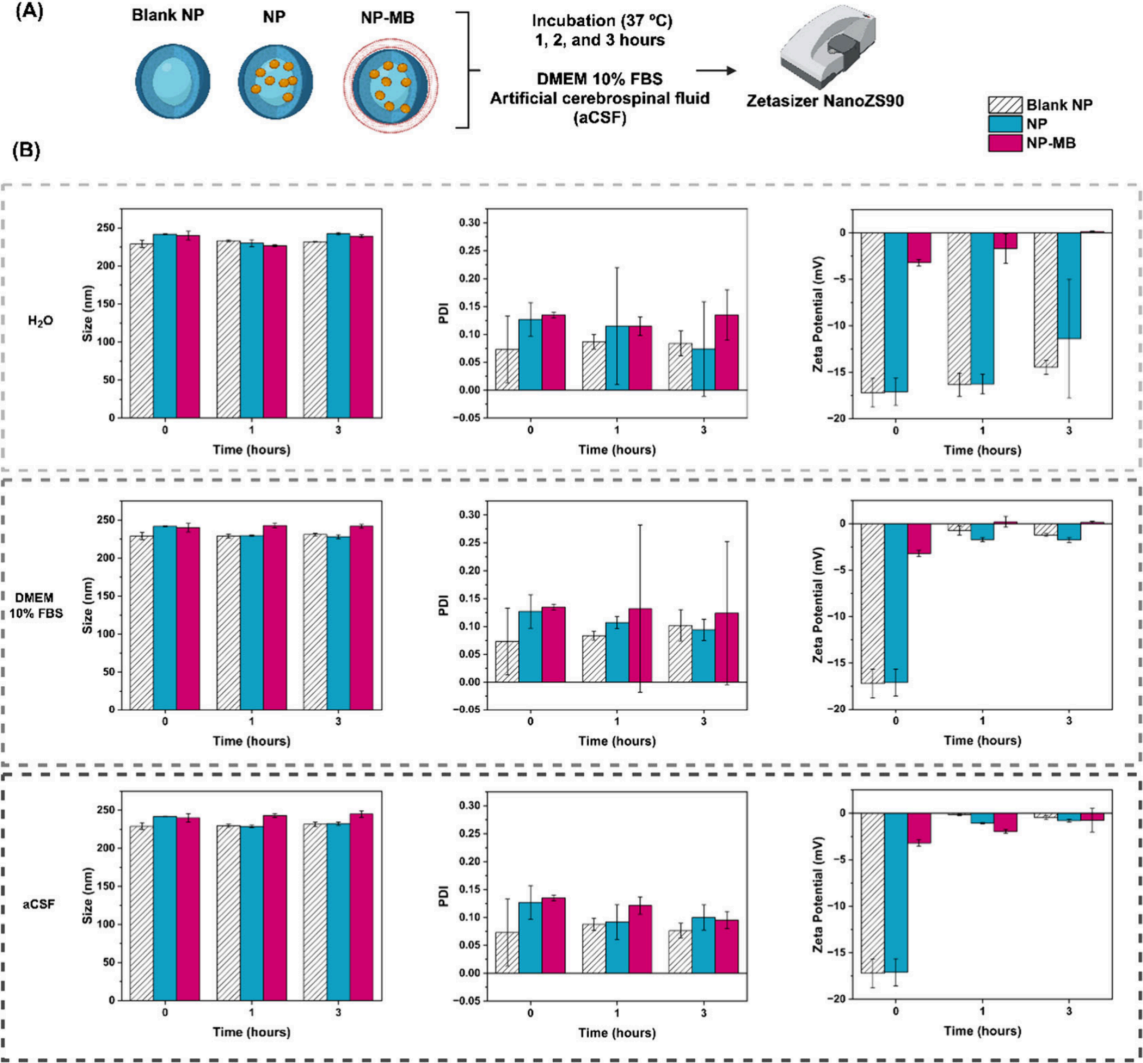
Analysis of protein corona (PC) formation for blank NP, NP, and NP-MB in different media. (A) Schematic representation of the conduct PC study. Created with BioRender.com. (B) size (nm); PDI and zeta potential (mV). Statistical analysis using two-way ANOVA was applied to identify differences over time (*p* < 0.05).

Most studies on brain targeting have not evaluated protein corona formation via this type of delivery route. However, as the nasal cavity and cerebrospinal fluid exhibit particularities that should be considered for nanoparticle performance, this evaluation is highly required.^26^ Therefore, we have evaluated PC formation in blank NP, NP, and NP-MB using DMEM + 10% FBS (to estimate the behavior of these nanosystems for *in vitro* assays) and artificial cerebrospinal fluid (aCSF), to investigate whether the soft corona formation could provide any physicochemical changes in terms of size, PDI and ZP. A schematic of the PC experiment is shown in Figure 2A. No significant changes were observed in the particle size or PDI of any of the analyzed nanostructures (Figure 2B). However, ZP showed significant changes after incubation with DMEM + 10% FBS and aCSF compared to the negative control (Figure 2B).

For both nanostructures, incubation with the cell culture medium and aCSF for the first hour led to an increase in their ZP values close to neutral, which also represented the values recorded for the isolated media (Figure 2B). In addition, no significant changes in ZP were observed between 1 and 3 h of incubation, suggesting that a soft corona could be formed after 1 h. According to Partikel and co-workers, protein adsorption on negatively charged PLGA NPs increases the ZP values close to neutrality. Furthermore, the formation of this soft corona did not follow the protein concentration.^27^

The stability of nanoparticle size in DMEM with 10% FBS, despite a shift in ZP toward neutrality, is probably due to protein-mediated stabilization.^28^ Proteins in the medium provide steric and electrostatic protection, effectively preventing nanoparticle aggregation. While ZP remains a significant factor, proteins in the environment can mitigate its effect by adsorbing onto the nanoparticle surface, modifying the surface chemistry, and providing stabilization that is independent of ZP.^28−30^ This highlights the importance of accounting for the biological environment in assessing nanoparticle behavior.

In summary, the analysis of PC formation after blank NP, NP, and NP-MB incubation with DMEM + 10% FBS and aCSF has shown no alterations in terms of size and PDI data compared to the negative control. However, an increase in their ZP values, which were initially negative and close to neutral, was observed. Notably, the nanoparticle coating applied to the isolated cell membrane (NP-MB) had no additional effect on PC formation compared to blank NP and NP.

### 2.3 TMZ Loading, Stability, and Release Profile

The TMZ loading content (EE%) was determined by the indirect method and drug quantification by HPLC-UV applying previously validated methodology.^20^ The results showed that EE% was 50 ± 14% and 45 ± 10% for NP and NP-MB, respectively. These values were confirmed by disrupting the NP core with an organic solvent and subsequent drug quantification. A previous study investigating the encapsulation of TMZ in the PLGA core highlighted some challenges and low efficiency.^31^ Herein, we explored different mechanisms that could improve the EE%. The double-emulsion method applied with the chosen solvent system has been identified as a promising strategy to optimize loading of TMZ into the PLGA core. Following this methodology, similar EE% indices have been proposed.^31^

Considering TMZ hydrolyzation under physiological conditions (pH 7.4) and the conversion to its active form MTIC,^32^ which loses its characteristic UV absorption, we performed a previous stability study of TMZ by analyzing its absorption in the UV spectrum after exposure to different media for release study (Figure S2). Our results confirmed that TMZ exposed to DMEM (pH 7.4) underwent immediate hydrolysis with a significant reduction in UV absorption at 330 nm. After 1 h, the UV absorption reduced by approximately 50% (Figure S2A). This can be attributed to the short half-life (approximately 2 h) of TMZ under physiological conditions.^31^

The exposure of TMZ to pH 6.5 minimized the hydrolysis process so that, in this case, the absorbance exhibited a small reduction after 24 h (Figure S2B) and just after 72 h, analysis reached half of the initial absorbance recorded. The performance of TMZ exposed to pH 5.5 in the presence or absence of ascorbic acid significantly improved the maintenance of TMZ molecules (Figure S2C and S2D). In addition, the use of ascorbic acid promoted a higher definition of the UV spectrum. This greater stability in an acidic environment has been previously reported.^33^ Therefore, we selected this medium to analyze the release and permeation profiles of the developed nanosystems (NP and NP-MB).

Dissolution tests for free TMZ, NP, and NP-MB were conducted using a Franz cell system with a synthetic membrane that is considered appropriate for topical dosages, including nasal products. This system, characterized by low volume of dissolution media and unidirectional drug diffusion across the membrane, closely mimics the nasal cavity conditions.^34^

**Figure 3:**
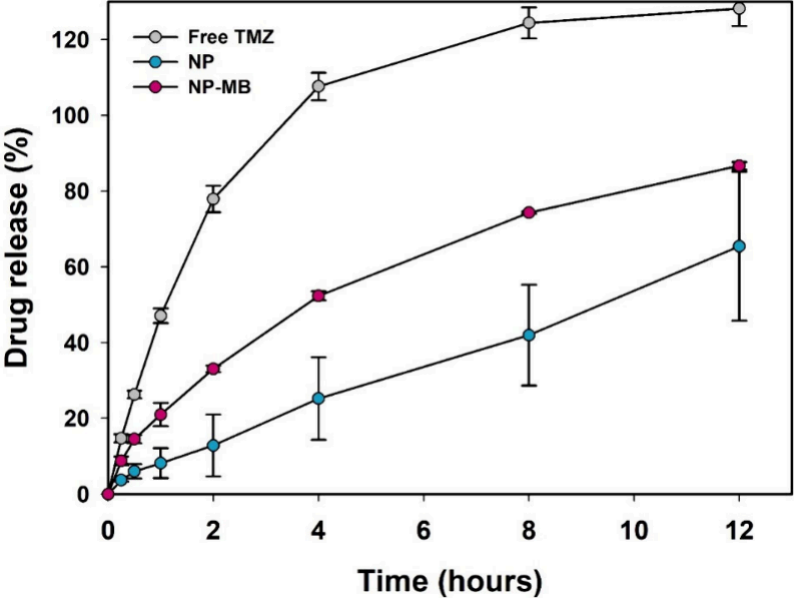
TMZ release profile (%) from NP (dark blue) and NP-MB (pink) in a phosphate buffer with 0.1% ascorbic acid, pH 5.5. Data shows the average of six measurements (*n* = 6) and their standard deviation (SD). Statistical analysis using one-way ANOVA with Tukey’s comparisons was applied to identify differences between experimental groups (*p* < 0.05).

The release profiles of the free drug and TMZ from NP and NP-MB are shown in Figure 3. The complete release of free TMZ occurred within 4 h, where 50%, 80%, and 100% release was achieved after 1, 2, and 4 h, respectively. In contrast, the encapsulation of TMZ into NP and NP-MB resulted in significantly lower release rates. After a 2 h assay, TMZ released from NP and NP-MB was 10% and 30%, respectively. In addition, after 4 h, the drug release was two times lower for NP-MB and five times lower for NP than for the free drug, a release pattern attributed to the effective entrapment of TMZ. Detailed analysis of NP and NP-MB suggested that the drug release of TMZ occurred more sharply in the first 4 h and subsequently slowed until 12 h. The release profile of TMZ from the PLGA core followed the profile of several hydrophilic drugs encapsulated in these nanostructures. To date, a dual-step behavior is commonly observed, where partial drug release occurs faster and partial sustained release occurs in the second step.^31^

Coating with an isolated cell membrane (NP-MB) enhanced drug release. Considering the conditions applied for membrane coating (sonication bath at low temperature), conformational changes in the polymer chains may be expected, which can facilitate drug diffusion into the polymeric network and, accelerate the release process.^34^ Therefore, the coating procedure may have increased PLGA chain flexibility, slightly accelerating TMZ release, which can be corroborated by the analysis of the acquired *k* values for NP-MB (Table 2). Parameter *k* represents a constant drug transport rate that is closely linked to the drug release kinetics from the nanostructured polymeric platform. Consequently, a higher *k* is directly related to faster release, whereas lower values are associated with poor drug release from nanocarriers.^35^

**Table 2:** Parameters Extracted from Mathematical Models Baker and Lonsdale, Hixson–Crowell, Higuchi, First Order, Korsmeyer–Peppas, and Weibull Applied to TMZ Acquired Release Profiles

Mathematical models		NP	NP-MB
**Baker and Lonsdale**	***r***^**2**^	0.872	0.967
	***k***	0.005	0.015
**Hixon–Crowell**	***r***^**2**^	0.989	0.967
	***k***	0.023	0.051
**Higuchi**	***r***^**2**^	0.902	0.991
	***k***	15.429	25.133
**First Order**	***r***^**2**^	0.985	0.988
	***k***	0.077	0.186
**Korsmeyer–Peppas**	***r***^**2**^	**0.995**	0.998
	***k***	7.392	21.966
	***n***	0.867	0.593
**Weibull**	***r***^**2**^	0.891	**0.999**
	***b***	0.438	0.764

To gain thorough insight into the mechanisms governing TMZ release from NP and NP-MB, we applied various mathematical models (Baker and Lonsdale, Hixson–Crowell, Higuchi, first order, Korsmeyer–Peppas, and Weibull) to the recorded drug release profile (Figure 3). Analysis was performed based on the coefficient of determination (*r*^2^). Kinetic parameters can offer insights into how the developed polymeric nanoplatform influences drug release.^36^

The TMZ released from NP and NP-MB correlated better with the Korsmeyer–Peppas and Weibull models (*r*^2^ = 0.995 and *r*^2^ = 0.999, respectively). The Korsmeyer–Peppas semiempirical model suggests that drug release phenomena are linked to drug diffusion and dissolution from the polymeric matrix.^36,37^ According to this model, drug release and elapsed time are exponentially related according to eq 1.^38^
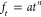
1where *a* is a constant that relates the structural and geometric characteristics of the polymeric platform; *n* represents the release exponent, and the function of *t* (fractional release of drug).^38^ Within this model, the *n* parameter helps translate the release mathematics into a mechanistic interpretation of the acquired data set, providing a deeper understanding of our findings.

In general, for spherical particles, such as NP nanostructures, *n* < 0.5 reveals the Fickian diffusion. Anomalous transport is considered between 0.5 and 1, where a combination of Fickian diffusion and swelling governs the event. For *n* = 1 or *n* > 1, the release is represented by case II transport or supercase II transport, respectively.^38^ Therefore, our data suggest that the release of TMZ from the NP samples follows anomalous transport. Similar PLGA nanoparticles with PVA as a stabilizing agent also exhibited the greatest correlation with Korsmeyer–Peppas, with a close release *n* exponent value.^39^

Originally delineated by Weibull in 1951, the Weibull equation proposes that the logarithm of the drug released and time should have a linear relationship.^40^ According to this model, the cumulative drug release at a pre-established time can be adjusted to different dissolution profiles according to eq 2.
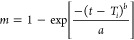
2where *m* is the accumulated drug in the medium, *a* is a time-dependent scale parameter that defines the process, *T*_*i*_ is the point parameter, *t* represents the lag time before the onset of the release process,and *b* describes the shape of the dissolution curve progression. Therefore, exponent *b* considers the entire data set and mechanism of diffusional release, indicating the systems that drive drug transport through the polymer matrix. For *b* values similar to those recorded in this study (*b* = 0.76), a combination of different mechanisms may govern the release process.^41^

Therefore, for NP-MB, TMZ release was initially governed by diffusion through the polymeric matrix once PLGA became a swellable network. Later, as the polymer is a polyester that can be hydrolyzed into soluble oligomers and further into monomers under physiological condition,^39^ ester bond hydrolyzation causes erosion of the matrix, allowing late drug release from the entrapped TMZ molecules.^42^

Collectively, the adopted strategy for greater EE% for NP and NP-MB has provided a great index of TMZ association (close to 50%). The results from the release profile showed that in the case of NP, TMZ release followed anomalous transport, whereas for NP-MB, TMZ was released by a combination of drug diffusion through the swellable matrix and erosion of the PLGA polymer.

### 2.4 *Ex Vivo* Permeation Study Applying Nasal Porcine Mucosa

**Figure 4:**
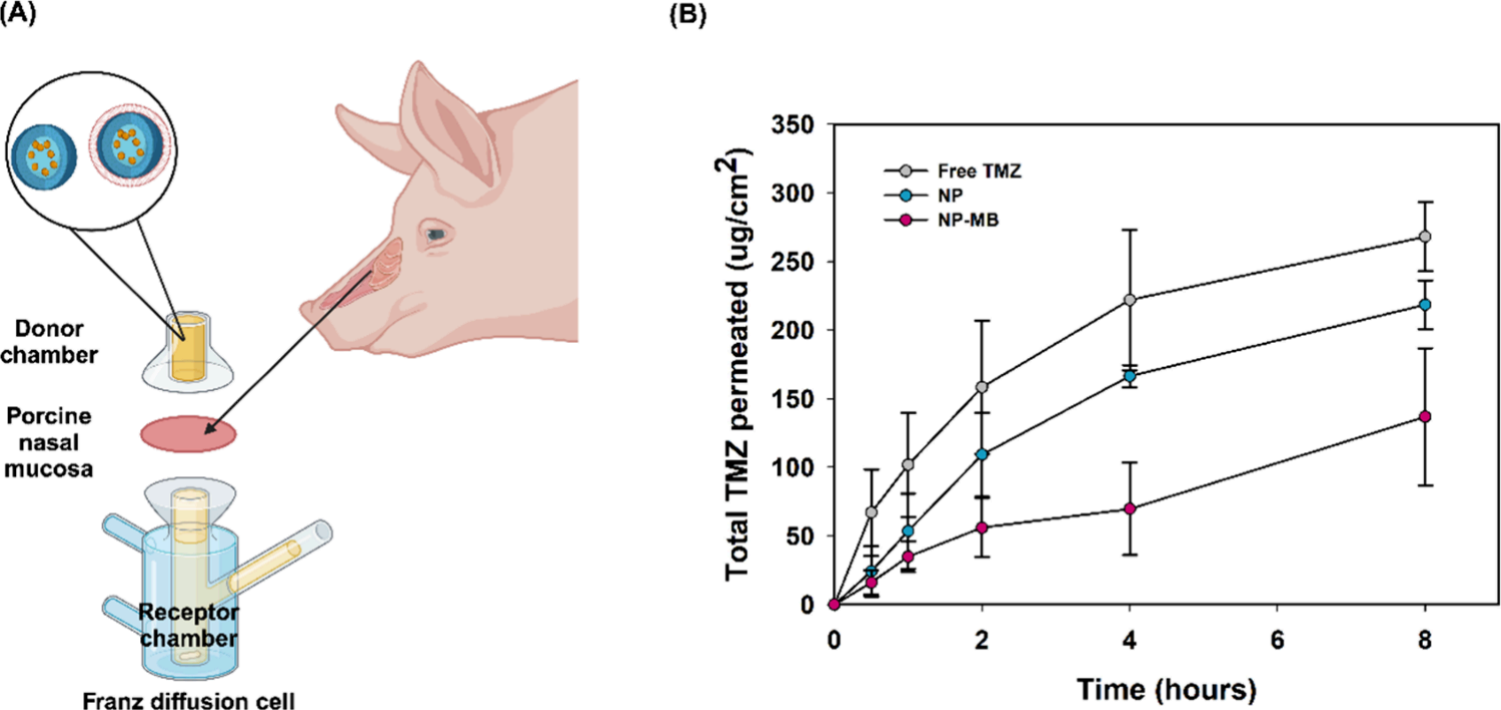
*Ex vivo* permeation study applying nasal porcine mucosa. (A) Schematic representation of *ex vivo* permeation study applying Franz diffusion cells. Created with BioRender.com. (B) Total TMZ permeated (ug/cm^2^) from free TMZ (gray), NP (blue), and NP-MB (pink) in a phosphate buffer with 0.1% ascorbic acid, pH 5.0. Statistical analysis using one-way ANOVA with Tukey’s comparisons was applied to identify differences between experimental groups (*p* < 0.05). Data show the average of six measurements (*n* = 3) and their standard deviation (SD).

Effective delivery via the nose-to-brain route relies entirely on the adequate permeability of the nasal mucosa. Ideally, the potential of polymeric nanostructured delivery systems to permeate nasal mucosa should be investigated in human tissues. However, considering the challenges regarding ethical and availability aspects, nasal porcine mucosa can be alternatively applied because of its similarity to human mucosa in terms of physiology, anatomy, histological, and biochemical aspects.^43^*Ex vivo* permeation of free TMZ, NP, and NP-MB was investigated by applying nasal porcine mucosa. A representative illustration of the *ex vivo* permeation assay is shown in Figure 4A.

According to the permeability profile in terms of total drug permeated (μg/cm^2^) against time, free TMZ showed a higher permeation profile (*p* ≥ 0.05) throughout the test with 250 μg·cm^–1^ drug permeated after 8 h (Figure 4B). Also, the permeability potential had a slight decrease from free TMZ to NP and a considerable decrease in NP-MB, where the total drug permeated after 8 h was 200 and 150 μg·cm^–1^, respectively. For all the samples, the permeation profile first exhibited an exponential increase, followed by a linear trend, which corresponded to unsteady and steady-state conditions.^44^

Drug crossing along the nasal epithelial membrane may occur via a transcellular route considering concentration gradients, receptor-mediated or vesicular transport, or a paracellular route through tight junctions.^44^ Drug molecular weight and lipophilicity greatly impact the permeability potential. As TMZ is an alkylating agent with a small and neutral structure at physiological pH and a great ability to cross the BBB, its higher permeability as a free drug was expected.^45^

For NP-MB biomimetic systems, the presence of an isolated U251 membrane associated with the PLGA core can result in physicochemical interactions with the porcine mucosa, promoting partial retention of these systems. Although the permeability potential of NP-MB was not ideal, this was one of the few studies that demonstrated the permeability potential of nanostructures coated with isolated cell membrane NPs using *ex vivo* protocols. The exploration of the nose-to-brain concept remains unaddressed, reinforcing the utmost importance of this assay. Importantly, the residence time of the system in the nasal cavity and mucosa directly affects its permeability. Therefore, efficient interaction of the nanostructure with the mucus layer in the nasal cavity could increase the retention time of the NPs and consequently enhance drug permeation.^46^

### 2.5 Cell Viability Assay and Cell Death

The IC_50_ values recorded for TMZ in malignant brain tumor cells, such as the culture strains applied to delineate this trial, were high, with a wide range of concentrations varying from micromolar to millimolar levels.^47^ The transposition of these values to biomimetic nanostructures would have resulted in the deposition of excessive NP over the cell monolayer, which may impair important physiological exchanges between the cells and the surrounding media (such as nutrients, metabolites, and O_2_ and cell waste), compromising their viability. Therefore, we have investigated undesired toxicity, performing a cell viability screening using 1.25 to 20 μL of NP per well (0.1–1.6 × 10^10^ particles/mL) of blank NP and NP since above 20 μL of NP, the culture medium became turbid possibly due to the nanoparticle aggregation compromising physiological exchanges between cells and the culture media.

**Figure 5:**
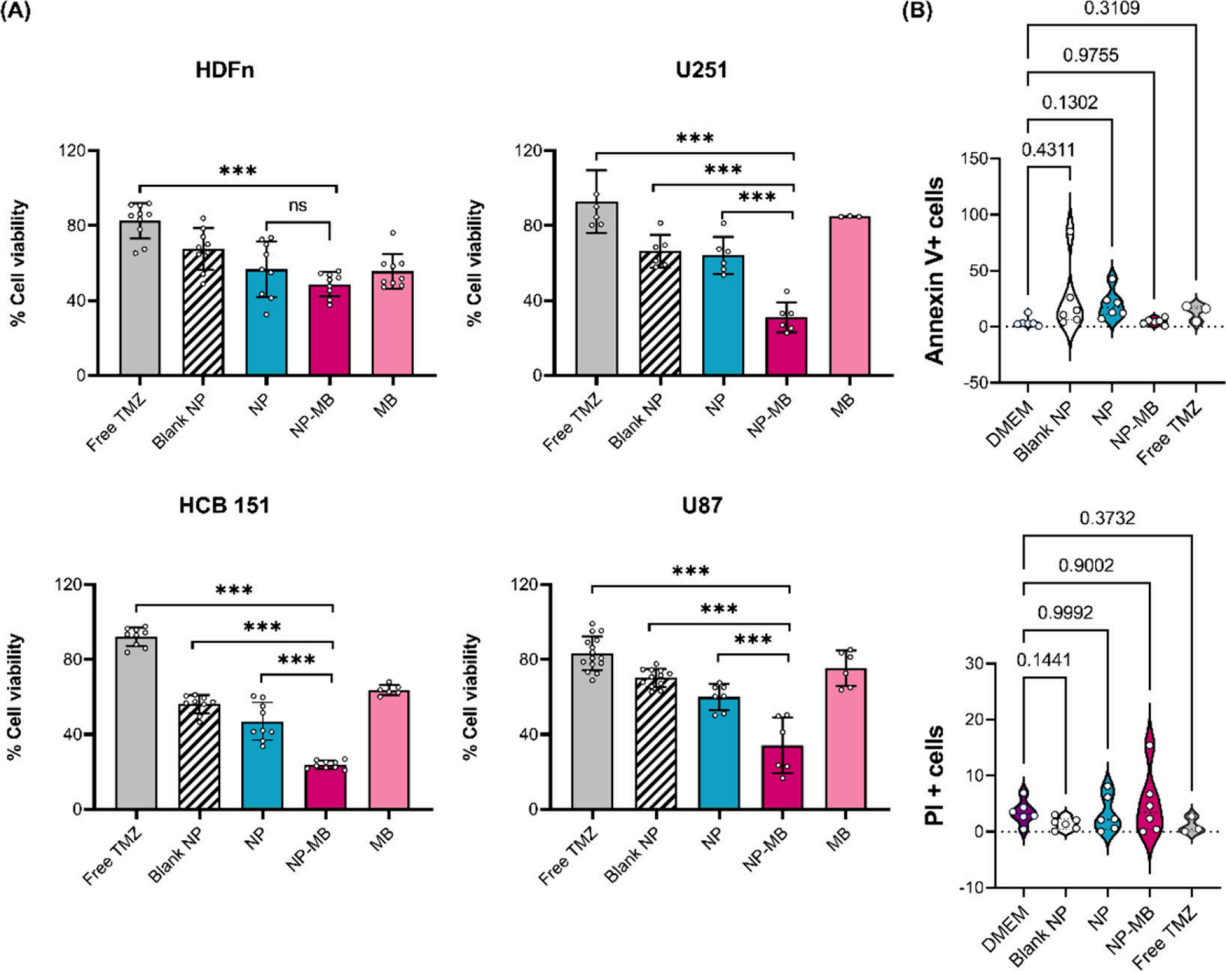
Cell viability assay and cell death. (A) Comparison of different treatments: free TMZ, blank NP, NP, and NP-MB in different cell lines (HDFn nontumoral, U251, U87, and HCB151). Results represent the median ± SD of at least 3 independent assays (*n* = 3). The following controls were applied: cells incubated with complete medium, cells incubated with complete medium and the same volume of DMSO in the TMZ groups, and cells incubated with complete medium and the same volume of water in the blank NP, NP, and NP-MB groups. Differences *p* < 0.01 between applied treatment were considered statistically significant (***). (B) Cell death was determined by Annexin V FITC and Propidium Iodide staining and flow cytometry after 72 h of treatment with either free TMZ, blank NP, NP, and NP-MB. Results are expressed as the percentage of Annexin + and PI + cells (*n* = 3) in relation to the control. *p*-values above the bar describe the trend from recorded applied treatment.

Our results have shown that when 0.1 × 10^10^ particles/mL of blank NP was applied as a treatment, no significant reduction in cell viability (approximately 80%) was observed for HDFn and U251 cells (Figure S3A). For U87 and HBC151, the same concentration of particles has provided 64 and 48% reductions in cell viability, respectively. Therefore, this fact reinforced the biocompatibility of the blank NP with human fibroblasts, which is desirable for nanopharmaceutical applications. Conversely, when we applied NP treatment (nanoparticles loaded TMZ), 0.1 × 10^10^ of NP promoted around 50% reduction in cell viability for all cells, an activity related to the TMZ drug encapsulated into the NP (Figure S3B). Considering the results for this initial screening, we applied the same concentration (1.25 μL/well) and transposed this amount for TMZ dosage for future viability evaluation comparisons between free TMZ, blank NP, NP, and NP-MB (Figure 5A).

In general, considering the concentration of TMZ encapsulated in the NP, the free drug (free TMZ treatment) did not significantly reduce cell viability in any of the analyzed cells. For HDFn, blank NPs did not significantly alter the cell viability. Interestingly, for all cells, treatment with blank NP and NP (NP loaded TMZ) did not affect cell viability (Figure 5A).

Considering the high IC_50_ values found for TMZ in distinct publications and the fact that the encapsulation efficiencies of hydrophilic molecules such as TMZ in PLGA nanoparticles are generally low, we state that in their current form, NPs would not promote a reduction in cell viability in an *in vitro* environment. However, we applied a coating strategy to improve the biological performance using the membrane isolated from the U251 cell (NP-MB).

Coating with isolated cell membranes reduced viability of all glioma cell lines (22 to 35%). In HDFn cells, the reduction in viability occurred to a lesser extent at only 50%. The decrease in viability recorded for the U251 source cell was significantly higher, where NP-MB provided 30% cell viability instead of the 65% supplied by NP treatment. These results indicate the successful coating of NPs with U251 cell membranes because the significant reduction in the viability of U251 cells was probably due to homotypic recognition, which may have allowed improved interaction of NP-MB with the parental cells. Therefore, significant targeting was observed when the NP-coated isolated cell membranes (NP-MB) matched those of their source cells (U251). Alternatively, a mismatch between nontumor cells and NP-MB resulted in weak binding.^16^

Notably, the viability assay recorded in HCB-151 cells, a patient-derived primary cell line, applying NP-MB treatment may provide a representative evaluation of biomimetic delivery system performance, confirming the specific homing preference of the parent cell. Although screening for different patient-derived cells should be performed further, the recorded toxicity for different glioma cells suggests the possibility of allogeneic treatment, which would benefit clinical translation.^48^

Collectively, the results for cell viability showed that the coating procedure using the extracted U251 cell membrane provided a significant improvement in terms of cell viability reduction for all tumor cell lines evaluated. Although these results support the homologous targeting capability of NP-MB against U251 source cells and other glioma cells, research must be carried out, especially to understand the mechanism behind this unique effect.

These results prompted us to further investigate how this biomimetic nanocarrier (NP-MB) promotes cell death compared with treatment applying free TMZ or TMZ encapsulated in PLGA NP. Gate strategies applied for cell death assay are depicted in Figure S4.

In living cells, phosphatidylserine (PS) is exclusively found in the inner cell membrane. However, during apoptosis, PS translocates from the inner to the outer side and should bind by the Annexin V providing fluorescence signals detected by flow cytometry.^49^ As depicted in Figure 5B, blank NP and NP provided higher expression of PS signaling apoptosis. In addition, the PS levels for free TMZ drug tended to decrease compared to those of NP, corroborating their lower potential to reduce cell viability in U251 cells. The reduction ability of free TMZ to trigger U251 cell death by apoptosis was dose-dependent; the concentration used in our study was lower than that previously reported.^50^ Previous studies have also shown the upper death potential of TMZ-loaded nanostructures compared to those treated with free drug.^51^ Importantly, the recorded results for PI+ provided evidence that treatment with NP-MB exhibits a higher potential to induce cell necrosis than every other treatment. This potential may correlate with its greater capacity to reduce the viability of U251 cells.

Induction of apoptosis is considered one of the primary strategies for cancer treatment. However, similar to several other complex regulatory pathways, the effectiveness of cancer treatments depends not only on the cellular damage that they cause but also on the ability of cells to activate their apoptosis program. Apoptosis is a double-edged sword because cancer cells may acquire resistance to standard therapies. Therefore, an improved strategy may initiate effective cell death at the early stage of treatment.^52^ From this perspective, the shift of cell-programmed death early triggered by the TMZ drug to the necrosis initiated from NP-MB may represent a significant advance in therapeutic efficacy. Altogether, the higher percentage of PI+ provided by NP-MB treatment led us to consider this biomimetic delivery system as a great alternative for initiating effective cell death in GBM therapy.

### 2.6 Cellular Internalization and Endocytosis Pathways of NP and NP-MB

**Figure 6:**
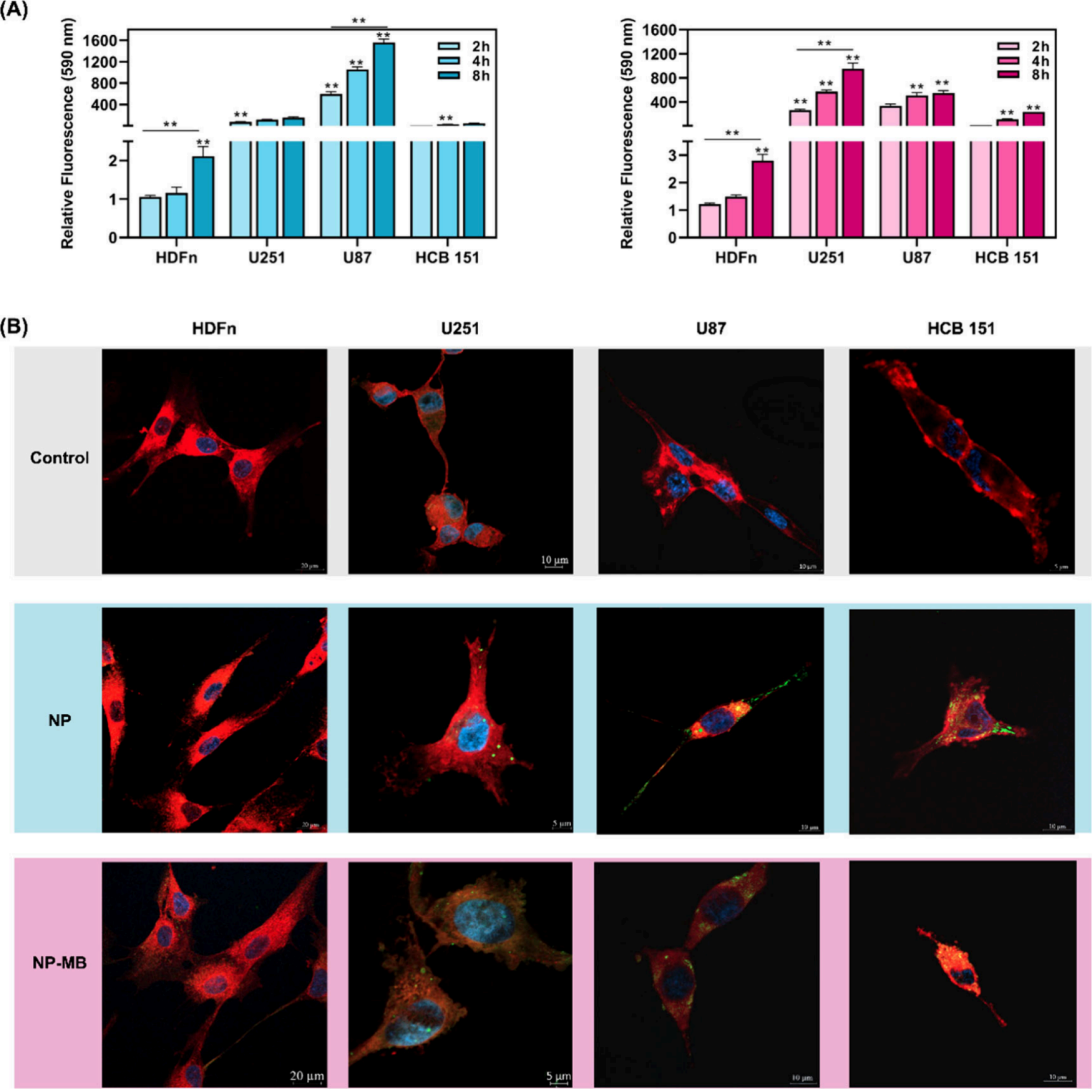
NP and NP-MB (10^10^ particles/mL) internalization in HDFn, U251, U87 and HCB151 cells. (A) Internalization kinetics of NP (blue) and NP-MB (pink) using Flow Cytometry. Results express the geometric mean of the fluorescence intensity and represent the mean ± SD of three independent replicates. Differences *p* < 0.05 between the control and applied treatment were considered statistically significant *p* < 0.05 (**). (B) Images were recorded using a Zeiss LSM 900 laser-scanning confocal microscope following 4 h treatment with NP and NP-MB.

We examined the cellular uptake ability of four cell lines: U251 (homologous line), HDFn, U87, and HCB151 to explore the ability of NP-MB to be recognized by the homologous cell line and show better cellular internalization than NP. We quantitatively measured the cellular uptake of NP and NP-MB at different incubation times using flow cytometry. Flow cytometry data showed that neither NP nor NP-MB was significantly internalized in HDFn cells until 8 h of incubation (Figure 6A). For U251 cells, NP internalization was not significant between 2, 4, and 8-h exposition, whereas for NP-MB, internalization was time-dependent and statistically significant after 4 h. In U87 cells, NP showed significant uptake after 4 h in a time-dependent manner. In contrast, NP-MB was internalized after 4 h; however, no statistically significant differences were found between 4 and 8 h. For HCB151 cells, NP and NP-MB internalization was significant after 4 h of incubation (Figure 6A). Upon analyzing the data from Figure 6A, we noticed that while U251 cells exhibited a significant time-dependent uptake of NP-MB, for U87 cells, this dependence occurred with NP nanosystems (without a cell membrane coating). Therefore, this shift reinforced the homotypic recognition between U251 cells and NP-MB.

Importantly, the biomimetic nanostructure built with a U251 isolated cell membrane (NP-MB) showed a greater affinity for U251 cells than for all other glioma cell lines (U87 and HCB151). After the 8 h assay, the internalization of NP-MB in U251 cells was almost two times greater than that in U87 cells and nearly four times greater than that in HCB151 cells. These data corroborated the greater potential of NP-MB to reduce the U251 cell viability, as discussed above. The potential of biomimetic functionalization of drug delivery nanostructures using extracted cancer cell membranes has been extensively applied to achieve greater specificity/targetability for tumor source cells.^53^

Images recorded by laser scanning confocal microscopy (Figure 6B) corroborate these results, reinforcing that the low potential for internalization of NP-MB in nontumor cells is equivalent to its high potential for homotypic recognition in U251 GBM cell source.

In summary, the internalization kinetics and images recorded for NP and NP-MB showed that the internalization of tumor cells (U251, U87, and HCB151) was significant after 4 h of exposure. In contrast, nanosystem internalization in nontumor cells (HDFn) occurred after only 8 h of exposure. The internalization of NP-MB by U251 cells was significantly higher than that of all other tumor cells, reinforcing the potential for homotypic recognition.

**Figure 7:**
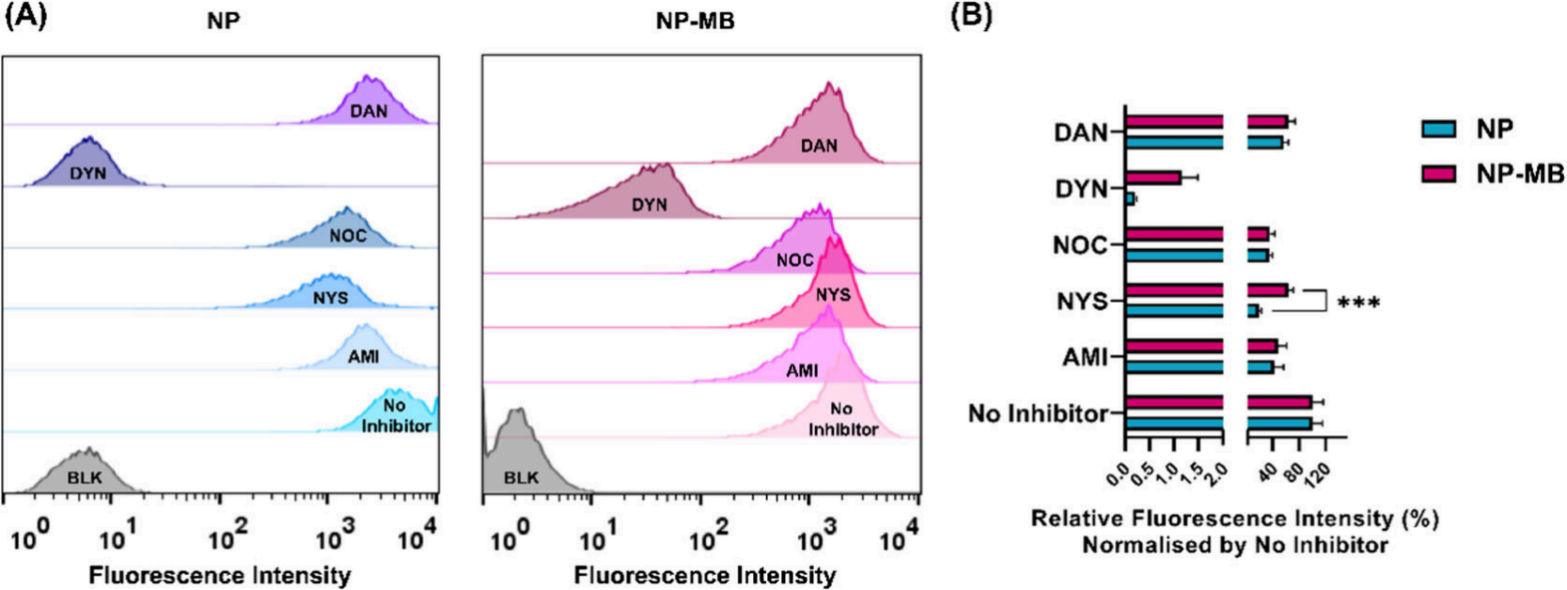
NP and NP-MB (10^10^ particles/mL) internalization mechanisms in U251 cells. (A) U251 cells were treated with different pharmacological endocytosis inhibitors amiloride (AMI), nystatin (NYS), nocodazole (NOC), dynasore (DYN), and dansyl-cadaverine (DAN) before incubation with NP and NP-MB (10^10^ particles/mL), for 4 h in the presence of the inhibitors. (B) Relative fluorescence intensity normalized by no inhibitor group. Results express the geometric mean of the fluorescence intensity and represent the mean ± SD of three independent replicates. Differences *p* < 0.01 between the control (no inhibitor) and applied treatment were considered statistically significant *p* < 0.05 (***).

We further investigated the mechanisms of particle internalization (NP and NP-MB) in U251 cells by applying different pharmacological inhibitors (for distinctive endocytic pathways). Endocytosis is an energy-dependent biological process that is responsible for the internalization of NP into eukaryotic cells. Understanding such mechanisms is essential to better predict how cells interact with these materials and to select their biomedical applications, because various endocytosis pathways are related to their biological effects and their possible undesired effects.^54,55^ As shown in Figure 7, NP and NP-MB are internalized by a combination of distinct uptake routes, because all applied inhibitors promote significant changes (reduction in fluorescence intensity) compared with the negative control (Figure S5).

For both systems, the use of dynasore (DYN) has resulted in a more than 90% reduction in cell uptake compared to the control, reinforcing that dynamin is an essential protein for NP and NP-MB internalization (Figure 7A). DYN, a cell-permeable small molecule, promotes noncompetitive inhibition of dynamin, a GTPase molecule. In eukaryotic cells, dynamin is one of the crucial regulators of endocytosis because it can be related to clathrin- and caveolin-mediated pathways, as well as some independent uptake mechanisms.^56,57^ Therefore, significant inhibition (more than 90%) was expected by the use of dynasore.

Cellular uptake of nystatin (NYS) mainly inhibited NP internalization, and the effect of nystatin on the formation of caveolae suggested that inhibition of the caveolin pathway primarily reduced the ability of the cell to transport NPs across the plasma membrane. Conversely, NP-MB uptake was less affected by the inhibition of this pathway (Figure 7B).

Cell treatment with nocodazole (NOC), a microtubule-depolymerizing drug, showed that NP and NP-MB may also require cytoskeleton machinery for their uptake. The results showed that exposure to NOC reduced the uptake of both nanosystems in a similar manner. Therefore, microtubules might play an essential role in cellular uptake.^58^ As a clathrin-, caveolin-, and dynamin-independent process, macropinocytosis mostly involves the uptake of larger particles.^57^ Herein, macropinocytosis may be considered for the NP and NP-MB uptake. Previously, cell treatment with amiloride (AMI) has provided uptake inhibition of around 50% of these nanosystems.

An investigation of nanoparticle internalization mechanisms in U251 cells showed that distinct internalization pathways are related to their uptake. Furthermore, in general, the inhibition potential provided by all the applied substances underscored similar results for NP and NP-MB. Among these, dynamin blockade proved to be the most significant for both nanosystems, with more than 90% inhibition of the ability of U251 cells to capture NP and NP-MB.

### 2.7 NP-MB Antitumor, Antiangiogenic Potential and Target Ability via the Nose-to-Brain Route

**Figure 8:**
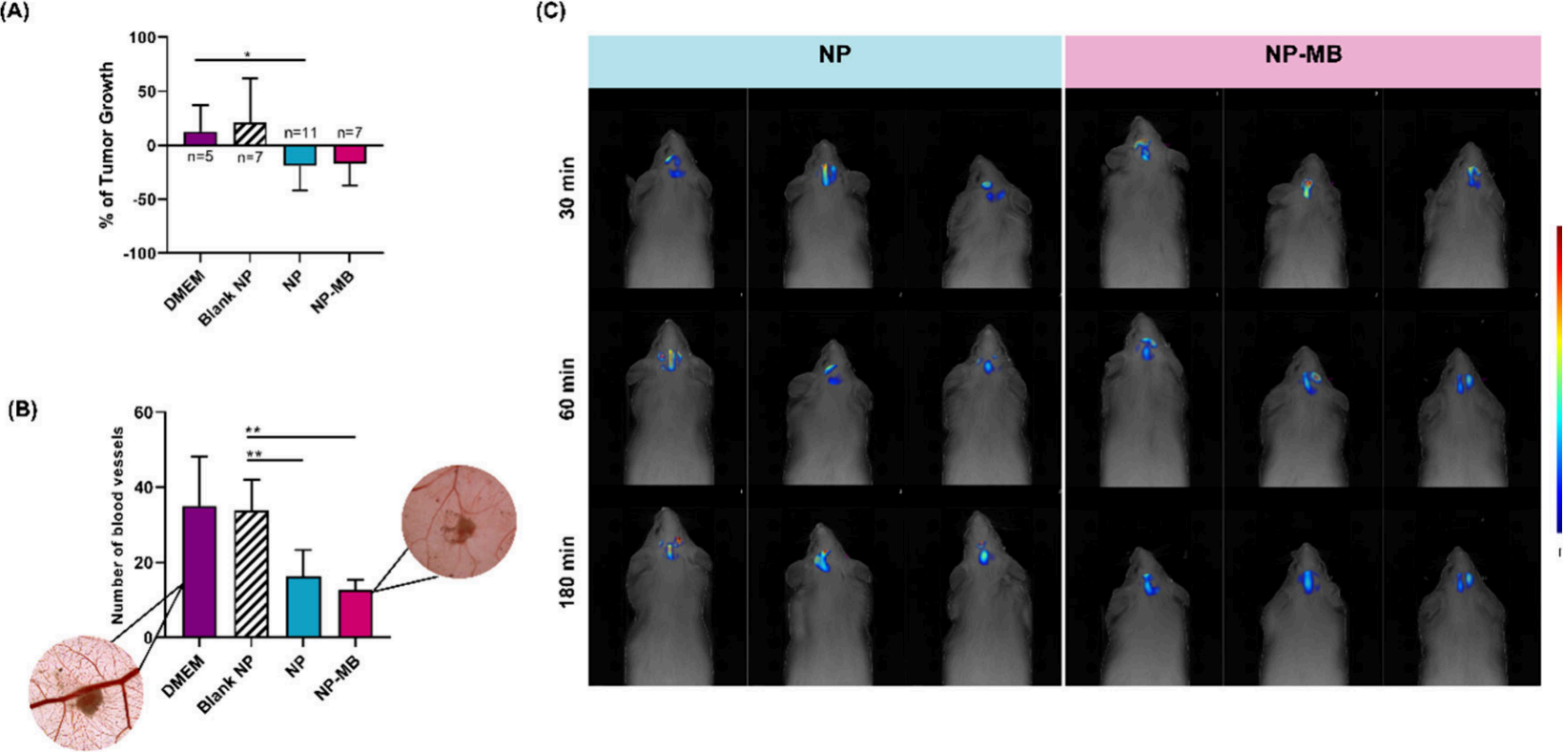
*In vivo* analysis for NP and NP-MB biological performance. (A) % of tumor growth after different treatments. (B) *Ex ovo* quantification of blood vessels number with representative images acquired 3 days after applying the treatment. Results are expressed as mean – SD. One-way analysis of variance, followed by Tukey’s multiple comparisons was used for statistical analysis **(*p* < 0.05). (C) Fluorescence tomography of the brain was conducted with images captured at 30, 60, and 180 min following the intranasal administration of IR780-loaded NP and NP-MB (*n* = 3).

The chorioallantoic membrane (CAM) assay is a powerful model for assessing biological performance, with a special focus on cancer biology due to its physiological relevance, and is considered a preclinical test needed to assess the potential of NPs for *in vivo* studies.^34^ Here, we employed the CAM assay to investigate the antitumoral and antiangiogenic activities, utilizing a 3D tumor model of U251 cells implanted into the CAM. Following tumor implantation and measurement, we categorized different experimental groups based on the recorded data from *in vitro* assays, including DMEM as a negative control, and blank NP, NP, and NP-MB. Tumor dimensions, such as area and perimeter, were quantified as a percentage of tumor growth, where each individual egg was considered as its own control (Figure 8A). The data revealed that the application of the negative control (DMEM) led to an approximate increase in tumor size (∼50%). Following the same trend, blank NP also demonstrated the capacity to promote tumor growth. Conversely, both NP and NP-MB exhibited equivalent levels of tumor dimension regression (∼15%).

The recorded *ex ovo* images (Figure 8B) demonstrated that treatment with DMEM and blank NP resulted in a significantly higher number of blood vessels, whereas the NP and NP-MB treatments resulted in substantial blood vessel reduction. Visual examination of the recorded *ex ovo* images 3 days after treatment revealed pronounced, adjustable, and larger vascular patterns in the negative control (DMEM). In the NP-MB treatment group, the vascular network exhibited anomalies, featuring a reduced and weak number of blood vessels (Figure 8B).

We also investigated the potential translocation of NP and NP-MB from the nasal cavity to the brain using *in vivo* FMT analyses. We observed the brain biodistribution of the formulations after the intranasal administration of IR-780-labeled nanostructures (Figure 8C). Tomography images showed fluorescent signals within 30 min of administration in all treated animals, with no visual differences observed between the NP and NP-MB groups. As previously reported, nanostructure potential uptake by the brain from the nasal mucosa can be achieved via two major pathways: a systemic pathway, which determines absorption into the blood circulation and then into the brain across the BBB, and a direct pathway, from the nasal mucosal epithelium into the brain, mainly along the olfactory or trigeminal nerves bypassing the BBB.^59^

Given the rapid acquisition of fluorescence signals, the nanostructures would have probably successfully translocated from the nasal cavity to the CNS.^60^ Preliminary studies by our group, along with published data on IR-780 release from polymeric PLGA nanocapsules, suggest that IR780 molecule release is minimal and occurs over an extended period.^61−63^ Based on the recorded time points, the accumulation of NP within cerebral tissue via the nose-to-brain route probably indicates nasal mucosa permeability. Furthermore, gradual fluorescence diffusion from the nasal area to the brain strongly supports a direct transport pathway as the primary mechanism. Although a systemic route via the nasal mucosa cannot be completely ruled out, neural pathways are assumed to be predominant. The exact mechanism of PLGA nanoparticle translocation from nose to brain remains unresolved and is a topic of ongoing debate in the literature.^64^

Although our *ex vivo* permeability results demonstrated that the biomimetic system faced challenges in permeating the nasal mucosa, the FMT results supported the hypothesis that both NP and NP-MB can permeate through the mucus barrier and be taken up by epithelial cells or neurons in the nasal cavity, ultimately reaching the brain. In addition, the recorded signals increased from 30 to 60 min and subsequently decreased to 180 min. The observed decrease in signal acquisition in this case might be related to the initial nanostructure degradation at the tumor site.

Systematic investigation must be conducted to further explain the nose-to-brain translocation potential of polymeric nanostructures. Nevertheless, our pilot study provided evidence that biomimetic nanoparticles can permeate the nasal mucosa and reach their target site of delivery in the CNS. To the best of our knowledge, experimental data on the ability of biomimetic nanostructures to reach the brain after IN administration have not yet been reported.

## 3 Conclusions

In this study, a novel biomimetic system, NP-MB, was established for efficient delivery of TMZ via the nose-to-brain route to treat GBM cells. The NP-MB is a highly versatile bioinspired nanoplatform modified with an isolated cell membrane to tailor specific interactions with source cells via homotypic recognition. In addition, the nanoplatform exhibited the ability to control TMZ release and promote TMZ permeation into the nasal porcine mucosa. According to *in vitro* analysis, the biomimetic system provided significant targeting to glioma cells, especially to their source cells. In addition, their potential to induce cell death reinforces their suitability for the therapeutic improvements required in GBM therapy. Furthermore, the *in vivo* data offer compelling evidence of the potential of NP-MB to induce tumor regression and exert antiangiogenic effects, in addition to emphasizing its ability to reach the brain, allowing nose-to-brain administration. To the best of our knowledge, this is the first study to apply TMZ loaded into bioinspired and biomimetic nanosystems intended for nose-to-brain delivery to improve GBM treatment. Although further work is required, especially to understand the mechanism underlying this unique effect using *in vivo* xenograft models, the results presented here indicate that NP-MB may represent a promising novel therapy for GBM treatment.

## 4 Experimental Section

### 4.1 Cell Lines and Cell Culture

Human glioma astrocytoma cells (U251), human brain glioblastoma astrocytoma cells (U87), and primary glioblastoma cell line (HCB151) derived from surgical biopsies, obtained in the Neurosurgery Department of Barretos Cancer Hospital^65^ (Sao Paulo, Brazil), acquired by the local ethics committee approval and the patient’s consent agreement, were kindly donated from Dr. Rui Manuel Reis, Barretos Cancer Hospital. According to the International Reference Standard for the Authentication of Human Cell Lines, cell authentication was performed through short tandem repeat (STR) DNA typing. The neonatal Human Dermal Fibroblast (HDFn) cell line obtained from Sigma-Aldrich (São Paulo, Brazil) was used as a nonmalignant cell line. All cell lines were grown in Dulbecco’s Modified Eagle’s medium (DMEM; Vitrocell Embriolife) supplemented with 10% fetal bovine serum (FBS), gentamicin sulfate (0.05 mg/mL), and amphotericin B (25 μg/mL), l-glutamine (0.584 mg/mL) at 37 °C in a humidified incubator, with an atmosphere of 95% air and 5% CO_2_.

### 4.2 NP and NP-MB Production/Synthesis

TMZ-loaded NP was produced by the double emulsion (W1/O/W2) method using TMZ drug (Sigma-Aldrich, Brazil), Poly(d,l-lactide coglycolide) (PLGA 85:15- Lactel Biodegradable Polymers), and poly(vinyl alcohol) (PVA, Sigma-Aldrich, Brazil) following previously described methodology.^20^ Blank NP was produced for comparative purposes. For internalization assays and confocal microscopy, 200 μL of 2 mg/mL 3,3′-Dioctadecyloxacarbocyanine perchlorate solution (DiO, Sigma-Aldrich) in dichloromethane was added to the organic phase before the NP sonication procedure. After the solvent evaporation, 2 mL of NP was added to Amicon 100-kDa cutoff, centrifuged at 800g using Eppendorf Centrifuge 5804R (Hamburg, GE) for 5 min (3 times), and stored for further use.

Image acquisition using fluorescence tomography was performed by mixing 200 μL of IR-780 (1 mg/mL) into the organic phase before NP sonication, resulting in IR780-labeled NPs. After preparation, the fluorescent NP underwent dialysis using a cellulose membrane with a 12 kDa cutoff (Sigma-Aldrich, USA) in PBS 7.4. The dialysis medium was changed iteratively, until no fluorescence signal was detectable.

To prepare cell membrane-coated NP (NP-MB), the cell membranes were extracted from the U251 cell line using a previously reported method.^66,67^ Briefly, U-251 cells were cultured in DMEM with 10% FBS until 80% confluence. Afterward, cells were detached from the cell culture flask and washed with ice-cold PBS. The cell pellet was resuspended in hypotonic and gradient buffers following extraction cycles. The disrupted cell dispersion was initially centrifuged to remove debris and nondisrupted cell organelles, and the supernatant was further ultracentrifuged for membrane precipitation.

For the coating procedure, 500 μL of NP (10^11^ particles/mL) and 500 μL of isolated MB (10^11^ particles/mL) were sonicated separately using a sonication bath (80 W potency and 37 kHz frequency) at 10 °C for 10 min and subsequently combined for additional cycle applying the same parameters.^20^

### 4.3 Physicochemical Characterization

Blank NP, NP, and NP-MB were characterized in terms of size, polydispersity index (PDI), and zeta potential (ZP) using Zetasizer Nano ZS (Malvern Instruments, Malvern, UK) equipment through photon correlation spectroscopy (wavelength 633 nm, 25 °C; 90° detection angle) and electrophoretic mobility, respectively. To evaluate more deeply blank NP and NP stability, considering the gap between the synthesis and membrane coating procedure, nanosystems were stored at 8 °C and analyzed for size, PDI, and ZP weekly. The analysis was conducted using the sample diluted in ultrapure water (100×). Results are expressed as the average of three independent measurements (*n* = 3) and their standard deviation (SD).

Concentration and size distribution were characterized using Nanoparticle Tracking Analysis (NTA) in a NanoSight NS300 (Malvern Instruments, Worcestershire, UK), equipped with a sample chamber and a 532 nm laser. The parameters camera level and particles per frame were maintained between 11/12 and 77 ± 25, respectively. All measurements were performed in independent triplicate (*n* = 3) at room temperature.

The morphological characterization of blank NP, NP, and NP-MB was carried out by negative-staining transmission electron microscopy (TEM) JEM-2100-JEOL 200 with a LaB6 source operating at an acceleration voltage of 200 kV. Samples were slowly dripped in a 400 Cu mesh carbon film TEM grid and stained using a 2% (w/v) uranyl acetate solution.^17^

Cryo-TEM analyses were carried out using Talos F200C (Thermo, USA), operating at 200 kV, equipped with a Ceta 16 M 4k × 4k camera (Thermo, USA) for digital image acquisition. For image acquisition, copper grids, lacey-type carbon film, and 300 mesh (#01895-F, Ted Pella, USA) for Electron Microscopy were used. The grids were treated with a 25 mA load for 50 s in EasiGlow (I) equipment (Ted Pella, USA). These grids were then fed to the Vitrobot Mark IV sample vitrification robot (Thermo, USA). The sample was applied, and the excess draining and grids were frozen immediately in liquid ethane. After this step, the grids were kept in liquid nitrogen until they were inserted under the microscope.

### 4.4 Protein Corona Formation

The stability of blank NP, NP, and NP-MB in the presence of proteins from different biological fluids was assessed by dynamic light scattering measurements using a Zetasizer NanoZS90, Malvern Instruments Ltd. Blank NP, NP, and NP-MB were first incubated (1:1) at 37 °C with ultrapure water as a negative control (absence of any salts and/or supplements that may affect the hydrodynamic size and/or the polydispersity), Dulbecco’s modified Eagle medium (DMEM) with 10% FBS and artificial cerebrospinal fluid (aCSF).^25^ Considering the reported translocation time related to the nose-to-brain transport for nanostructured systems intended for brain disorders, measurements were performed after 1, 2, and 4 h of incubation. After incubation, samples were diluted in ultrapure water (50×) and measurements were carried out at 37 °C, keeping attenuation of the samples during measurement at 11. The size, PDI, and ZP data represent the mean ± SD of the three independent measurements.

### 4.5 TMZ Loading, Stability, and Release Profile

TMZ encapsulation efficiency (EE%) was quantified using an indirect method, considering the free drug not encapsulated into the PLGA core according to eq 3. Initially, NP was added to Amicon filter 100-kDa cut off, centrifuged (8000 rpm 25 °C, 10 min), and the solution deposited on the bottom compartment was quantified applying high-performance liquid chromatography with a UV detector (HPLC-UV, Waters Alliance) following previously published methodology^68^ adapted and revalidated by us.^20^
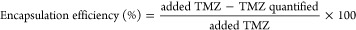
3

The chromatographic HPLC system used was Waters Alliance equipment with a quaternary pump and a Gemini NX-C18 column (250 cm × 4.6 mm, 5 μm, 110 Å, Phenomenex). The mobile phase consisted of acetic acid 0.5%: methanol (70:30, v/v) at a flow rate of 0.9 mL/min, with detection at 330 nm using a UV detector. The standard analytical curve was established by preparing a TMZ stock solution (100 μg/mL) in 0.5% acetic acid and further diluting it to the linearity range (5 to 50 μg/mL), resulting in the equation *y* = 74177*x* – 1660.6 (*r*^2^ = 1). Results are presented as the mean of three independent determinations with their respective standard deviations.

Considering the instability of TMZ in biological fluids, a previous study was carried out in Dulbecco’s Modified Eagle Medium (DMEM) pH = 7.4, phosphate buffer pH 6.5, phosphate buffer pH 5.5, and phosphate buffer with 0.1% ascorbic acid pH 5.0 to rationally select the medium to perform release and permeation studies. For this assay, a stock solution of TMZ in DMSO (128 μg/mL) was diluted in different media, and a scan in the UV–vis (Hitachi U2900) spectrum (200–600 nm) after preparation (*t*_0_) followed by 1, 2, 4, 6, 24, and 72 h stability. Results are the mean of three independent determinations (*n* = 3).

Release studies of free TMZ, NP, and NP-MB were performed according to a methodology previously proposed using a Franz diffusion cell (Microette-Hanson Research, Chatsworth, CA, USA).^34^ Cellulose membranes (D9402–100FT, avg. flat width 76 mm/3 in., Sigma-Aldrich, USA) were steady between donor and receptor chambers, and phosphate buffer with 0.1% ascorbic acid pH 5.0 stirred at 300 rpm, at 37 ± 0.5 °C, was used as the dissolution media. TMZ saturation in the receptor solution was evaluated to ensure the *sink* conditions. A known amount of TMZ solution, NP, and NP-MB was added to the receptor compartment and at predetermined times (15, 30, 60, 120, 240, 480, 720 min), aliquots were withdrawn, and quantification was performed using high-performance liquid chromatography and standard analytical curve constructed in the receptor media (*y* = 46.448*x* + 5.4553; *r*^2^ = 0.9995).

The release dates obtained were subjected to fitting with various mathematical models, including Korsmeyer Peppas, Higuchi, first order, Hixson–Crowell, Baker–Lonsdale, and Weibull. This analysis was conducted using SigmaPlot 10.0 software to gain insights into the mechanisms underlying the release of TMZ from both NP and NP-MB.

### 4.6 *Ex Vivo* Permeation Study with Nasal Porcine Mucosa Model

The evaluation of drug permeation profiles for free TMZ, NP, and NP-MB was conducted using porcine nasal mucosa obtained from a local slaughterhouse. The nasal mucosa was harvested immediately upon the animal’s euthanasia and frozen at −20 °C. Before the experiments, the mucosa underwent rehydration and temperature equilibration in the receptor medium to ensure tissue stability and sectioned for adjustment between the donor and receptor compartments of a Franz diffusion cell (Microette Plus, Hanson Research, Chatsworth, USA), with the mucosal surface precisely aligned with the donor compartment. The receptor chamber, containing 7 mL of a phosphate buffer with 0.1% ascorbic acid (pH, 5.0), was maintained at 37 °C and stirred at 300 rpm.

A known amount of TMZ, NP, and NP-MB was introduced into the donor ring. At predetermined intervals (30, 60, 120, 240, and 480 min), 2 mL of the receptor fluid was withdrawn and replaced with an equivalent volume. Each sample was analyzed in triplicate (*n* = 3) for comparative purposes.

### 4.7 Cell Viability Assay

The potential cytotoxicity of free TMZ, blank NP, NP, and NP-MB to HDFn, U87, U251, and HCB151 cells was evaluated by the MTT assay.^31^

For NP cytotoxicity, considering that human glioma cell lines mainly exhibit high IC_50_ values for TMZ and the fact that drug entrapment efficiency is generally low for TMZ into PLGA core, we would have to add an excessive number of blank and TMZ loaded NP to achieve the same concentrations tested for free TMZ. Many particles would sediment over the cell’s monolayer, hindering O_2_ and nutrient exchange between the cells and the surrounding media.^31,33^ Therefore, to avoid this unwanted cytotoxicity, we have initially performed a preliminary cell viability screening of the concentration in terms of particle/mL (from 10^9^ to 1.6 × 10^10^). After analyzing the data and selecting the ideal particle concentration, we used the same NP TMZ concentration for the free TMZ dosage and evaluated the comparative cell viability. The following controls were also performed: cells incubated with complete medium, cells incubated with complete medium and DMSO for the TMZ group, and cells incubated with complete medium and the same volume of water for blank NP, NP, and NP-MB groups. At the end of the exposure period, the culture medium was replaced by MTT and incubated for formazan crystal formation. Absorbance was then measured at 570 nm using a microplate reader (Spectra Max M3, Molecular Devices).

### 4.8 Cell Death

Dead Cell Apoptosis Kit assessed cell death (apoptosis/necrosis) with Annexin V FITC and Propidium Iodide (PI, ThermoFisher) according to the manufacturer’s instructions. The percentage of Annexin and PI positive events was measured by flow cytometry (FACS Calibur). Recorded results were analyzed using FlowJo.

### 4.9 Cellular Internalization and Endocytosis Pathways of NP and NP-MB

NP and NP-MB internalization studies were performed by labeling both nanostructures with the fluorescent dye DiO. HDFn, U87, U251, and HCB151 cells were seeded in 12-well plates at 4 × 10^4^ or 8 × 10^4^ cells/well and adhered overnight in 37 °C and 5% CO_2_ incubator. Afterward, cells were treated with 1 × 10^10^ particles/mL, for 2, 4, and 8 h, washed, and processed for flow cytometry analysis. The fluorescence intensity of NP and NP-MB was quantified in each sample by flow cytometry using FACS Calibur (BD Biosciences) and performed as three independent biological replicates (*n* = 3). Data were processed using FlowJo software, followed by one-way ANOVA for statistical significance analysis using GraphPad Prism software version 8.0 (GraphPad Software Inc.).

NP and NP-MB internalization was further confirmed by laser scanning confocal microscopy. Cells were grown on glass coverslips placed in 12-well plates and incubated with 5 × 10^9^ particles/mL DiO-labeled NP and NP-MB, for 4 h. At the end of the incubation period, cells were incubated with Cell Mask Deep Red plasma membrane stain (ThermoFisher) following the manufacturer’s protocol. Then, cells were fixed with 2% and 4% paraformaldehyde and stained with Hoechst 33342 (ThermoFisher). Finally, the coverslips were mounted with a Fluoroshield medium. Cells were imaged using a Zeiss LSM900 laser-scanning confocal microscope (Germany).

To investigate the endocytosis pathways involved in NP and NP-MB uptake in U251 cell line, cells were seeded in 12-well plates and preincubated with pharmacological inhibitors of different endocytic pathways (amiloride 100 μg/mL, nystatin 40 μg/mL, nocodazole 5 μg/mL, dynasore 100 μmol/L and dansyl-cadaverine 100 μg/mL), for 30 min, at 37 °C and 5% CO_2_. After the pretreatment, cells were incubated with DiO-labeled NP and NP-MB (10^10^ particles/mL) for 4 h. Finally, cells were thoroughly washed with cold PBS, harvested, pelleted in a complete culture medium, and processed for flow cytometry.

### 4.10 *In Vivo* Assays

#### 4.10.1 Analysis of Antiangiogenic Activity, Tumor Development, and Progression Using the Chicken Chorioallantoic Membrane (CAM) Assay

The antiangiogenic activity, tumor development, and progression were performed using fertilized chicken eggs provided by the local farm Criatório Mario Salviato (Porto Ferreira, SP, Brazil). These eggs were thoroughly cleaned with a 70% (v/v) ethanol solution and then incubated (Luna 480 Automatic Digital incubator, Chocmaster) in a humidified environment (70%) at 37 °C. At day 3 of development, a small window was made at the top of the eggshells by thoroughly removing shell fragments. The windows were sealed with adhesive tape to avoid dehydration and returned to the incubator under initial conditions.^69^ On day 9 of development, tumor cells, obtained from a suitable U251 cell line, were harvested, and a suspension containing 2 × 10^6^ cells was carefully applied onto the CAM surface using Geltrex matrix (LDEV-Free, hESC-Qualified, Reduced Growth Factor Basement Membrane Matrix, Thermo Fisher Scientific) through the window. On day 13 of development, after tumor grafting, pictures were taken to evaluate the tumor area and perimeter using the Leica M165C Microsystems Stereomicroscope coupled with a Flexacam C3 camera and LASX software. Eggs were then separated into different experimental groups, and treatments (Free TMZ, NP, and NP-MB) were applied. On day 16 of development, pictures were registered for the area and perimeter measurements, and CAMs with tumors were excised and photographed for blood vessel quantification purposes.^10^

#### 4.10.2 Validation of Nose-to-Brain Delivery Using Fluorescence Tomography

Male Swiss mice weighing 25 to 30 g were provided from the Central Animal Facility at the Federal University of Goiás (UFG). The mice were maintained in a controlled environment with a 12:12-h light–dark cycle at 25 ± 1 °C. They had unrestricted access to food and water and were allowed a one-week acclimatization period before the commencement of the experiments. *In vivo* studies received approval from the UFG Animal Research Ethics Committee (protocol 48/18). All experimental procedures adhered to animal care regulations and complied with Brazilian legislation, particularly Law 11,794 dated October 8, 2008. Brain delivery of the formulations after intranasal administration was evaluated by fluorescence tomography (FMT1500, PerkinElmer, USA). The animals were divided into 03 groups: negative control (no treatment was applied), NP and NP-MB (*n* = 3). Each group received into the nostrils 20 μL of treatment (corresponding to 1.6 × 10^10^ particles/mL) using a micropipette with the nonanesthetized animals kept in a supine position. The live brain fluorescence images were observed at 30, 60, and 180 min after administration of the formulations.^34,70^

## 5 Statistical Analysis

All tests were performed as three biologically independent experiments. Statistical treatment between experimental groups was compared using one-way analysis of variance, ANOVA, followed by the Tukey post hoc test using GraphPad Prism 8. Results were shown as a mean ± standard deviation and *p* < 0.05 was selected for statistically significant differences.

## Data Availability

The authors state that the data supporting the findings of this study are included in the article and its Supporting Information or can be obtained from the corresponding authors upon reasonable request.

## Supporting Information Available

Representative images of blank NP, NP, isolated membrane (MB), and NP-MB from cryo-TEM; analysis of TMZ stability in different media; cell viability initial assay; gate strategy applied for cell death assay; NP and NP-MB (10^10^ particles/mL) internalization mechanisms in U251 cells; representative ex vivo brain fluorescence tomography images taken for negative control (PDF)
