# Supplementary material for: Nose-to-Brain Delivery of Biomimetic Nanoparticles for Glioblastoma Targeted Therapy
Source: ACS Appl Mater Interfaces. 2024 Dec 18;17(1):484–99. doi: 10.1021/acsami.4c16837 (PMC11783514; doi:10.1021/acsami.4c16837)
Supplement: Supplementary file 1 — am4c16837_si_001.pdf [file am4c16837_si_001.pdf]

SUPPORTING INFORMATION

NOSE-TO-BRAIN DELIVERY OF BIOMIMETIC NANOPARTICLES FOR THE GLIOBLASTOMA TARGETED THERAPY

Natália Noronha Ferreira[a*]; Celisnolia Morais Leite[a]; Natália Sanchez Moreno[a]; Renata Rank Miranda[a]; Paula Maria Pincela Lins[b]; Camila Fernanda Rodero[a]; Edilson de Oliveira Junior[c]; Eliana Martins Lima[c]; Rui M. Reis[d,e] and Valtencir Zucolotto[a*]

[a] Nanomedicine and Nanotoxicology Group, *Physics Institute of São Carlos, São Paulo University*. Avenida Trabalhador São Carlense, 400, São Carlos, SP, 13560-970, Brazil.

[b] Hasselt University, Faculty of Medicine and Life Sciences, Biomedical Research Institute (BIOMED), Agoralaan, 3590 Diepenbeek, Belgium.

[c] Laboratório de Nanotecnologia Farmacêutica e Sistemas de Liberação de Fármacos, FarmaTec, Faculdade de Farmácia, Universidade Federal de Goiás - UFG, 5ª Avenida c/Rua 240 s/n, Praça Universitária, Goiânia, GO, 74605-170, Brazil.

[d] Molecular Oncology Research Center, Barretos Cancer Hospital, Rua Antenor Duarte Villela, 1331, Barretos, SP, 14784-400, Brazil.

[e] Life and Health Sciences Research Institute (ICVS), School of Medicine, University of Minho, Campus de Gualtar, 4710-057 Braga, Portugal.

*Email: noronhanat@hotmail.com / natalia.noronha@usp.br (N. N. Ferreira), zuco@ifsc.usp.br (V. Zucolotto)

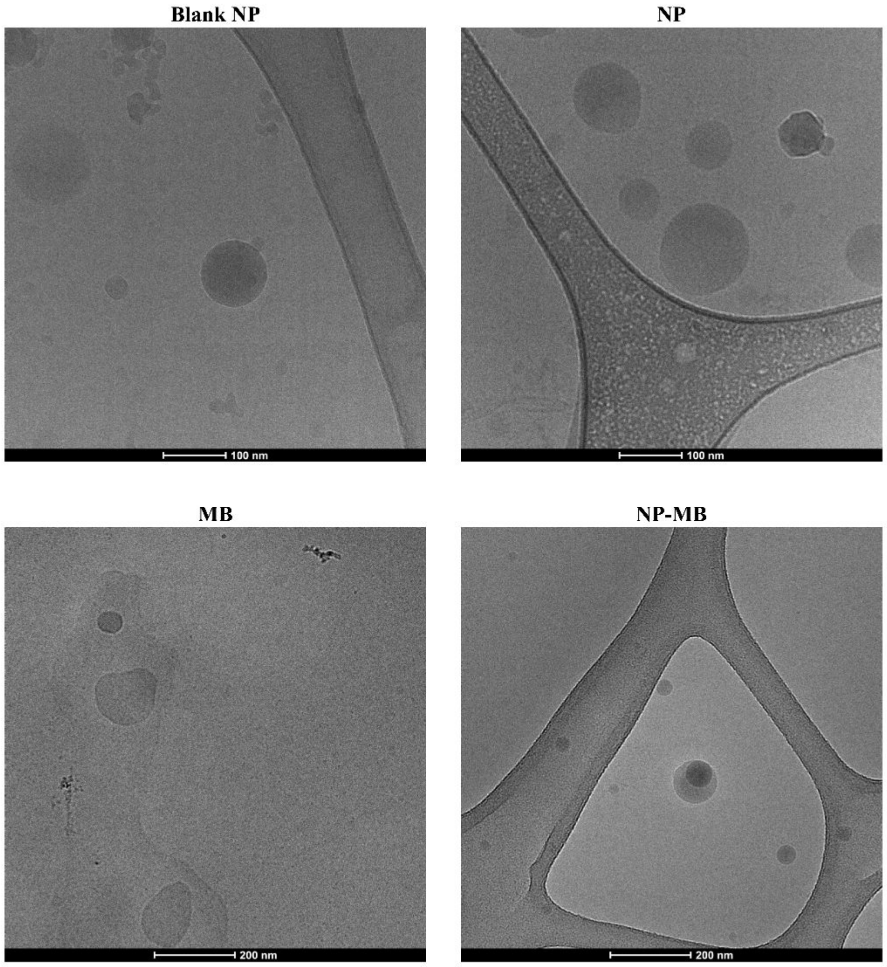

Figure S1: Development and characterization of NP-MB. Representative images of blank NP, NP, isolated membrane (MB), and NP-MB from cryo-TEM. Images were recorded using Talos F200C (Thermo, USA), operating at 200kV, equipped with a Ceta 16M 4k x 4k camera (Thermo, USA) for digital image acquisition.

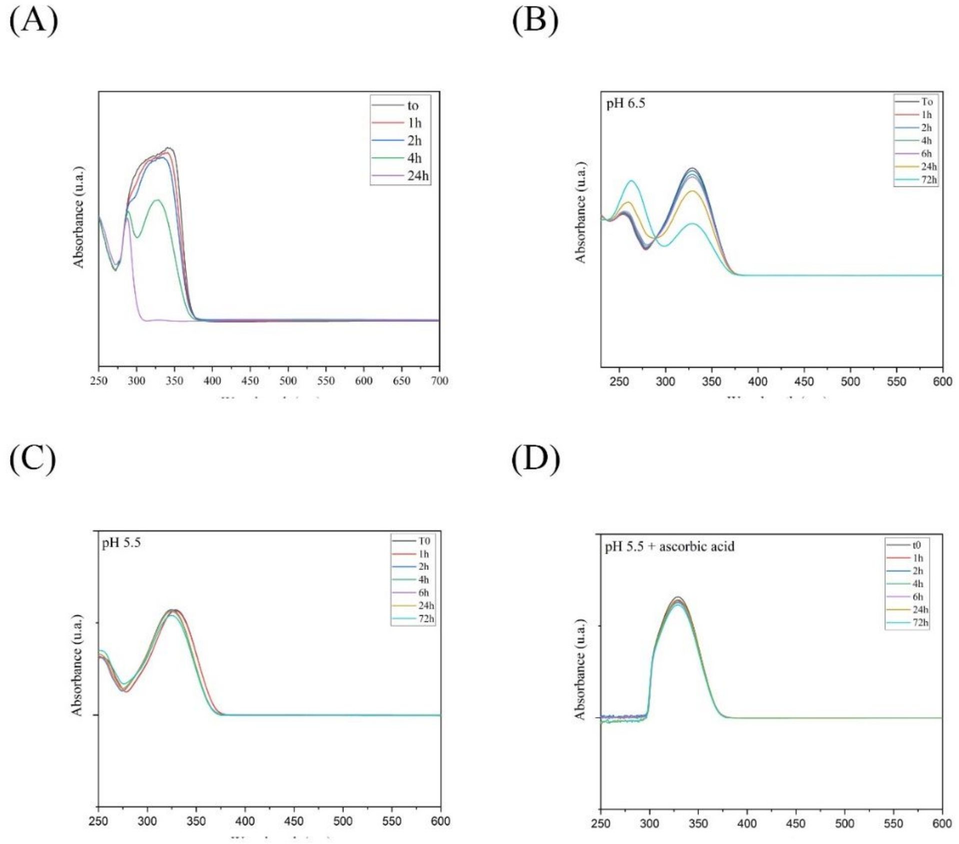

Figure S2: Analysis of TMZ stability in different media. (A) Dulbecco's Modified Eagle's medium – DMEM (pH 7.4); **(B)** Phosphate buffer pH 6.5; **(C)** phosphate buffer pH 5.5 and **(D)** Phosphate buffer with 0.1% ascorbic acid pH 5.0. Results represent the median of 3 independent measurements (n=3).

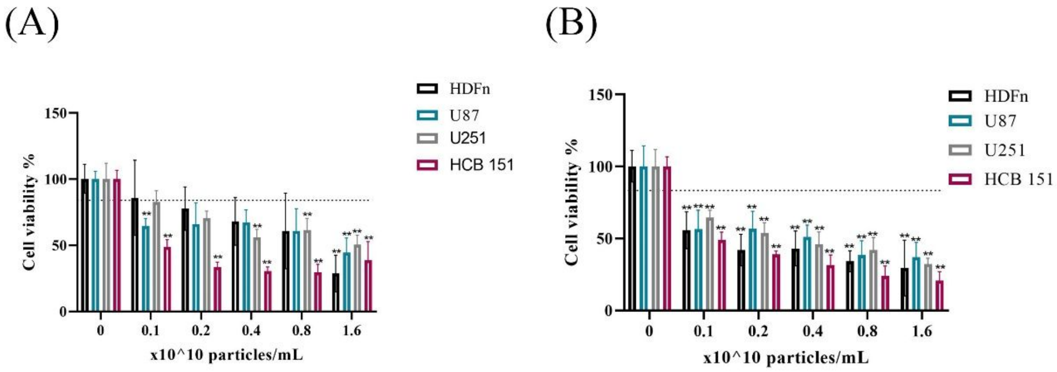

Figure S3: Cell viability initial assay. Screening of cell viability using 20 to 1.25 µL of NP per well (1x10^{9} to 1.6x10^{10} particles) applying **(A)** blank NP and **(B)** NP as treatment in different cell lines HDFn - non-tumoral, U251, U87, and HCB151). Results represent the median and SD of at least 3 independent assays (n=3). Differences $p < 0.05$ between the control and applied treatment were considered statistically significant $p < 0.05$ (**).

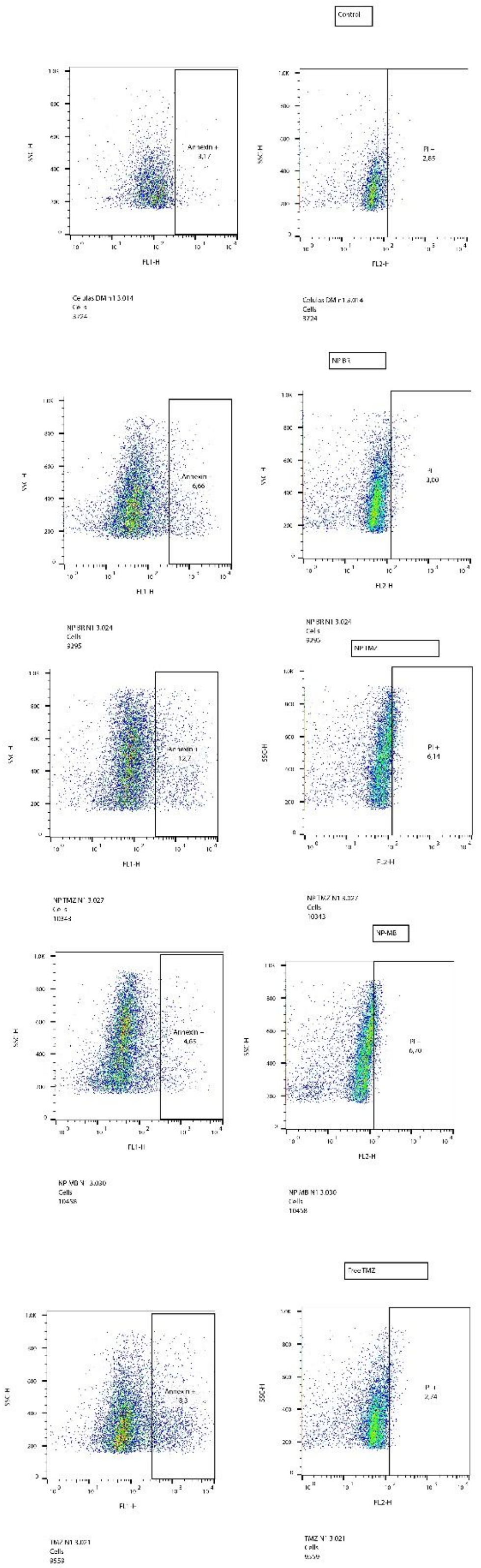

Figure S4: Gate strategy applied for cell death assay using Annexin V FITC & Propidium Iodide staining and flow cytometry after 72 h of treatment with either free TMZ, blank NP, NP, and NP-MB.

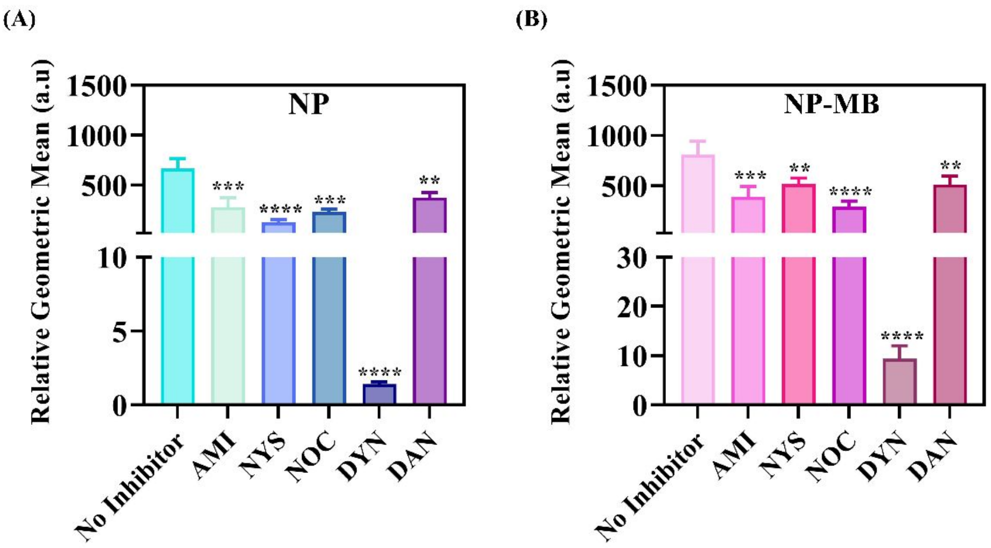

Figure S5: NP and NP-MB (10^{10} particles/mL) internalization mechanisms in U251 cells. U251 cells were treated with different pharmacological endocytosis inhibitors amiloride (AMI), nystatin (NYS), nocodazole (NOC), dynasore (DYN), and dansyl-cadaverine (DAN) before incubation with **(A) NP** and **(B) NP-MB** (10^{10} particles/mL), for 4 hours in the presence of the inhibitors. Differences $p < 0.05$ between the control (no inhibitor) and applied treatment were considered statistically significant $p < 0.05$ (**).

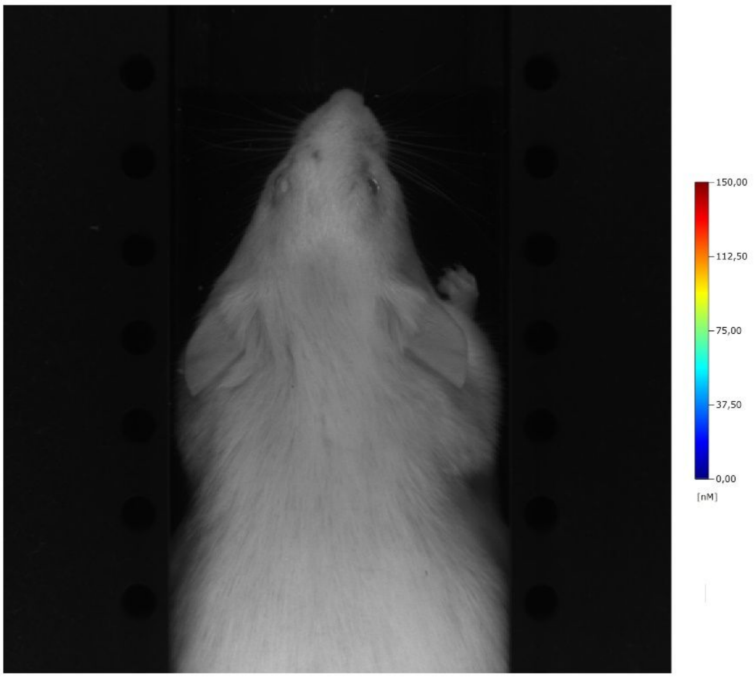

Figure S6: Representative *ex vivo* brain fluorescence tomography images taken for negative control (no treatment was applied).
